# The Home Learning Environment in the Digital Age—Associations Between Self-Reported “Analog” and “Digital” Home Learning Environment and Children’s Socio-Emotional and Academic Outcomes

**DOI:** 10.3389/fpsyg.2021.592513

**Published:** 2021-03-25

**Authors:** Simone Lehrl, Anja Linberg, Frank Niklas, Susanne Kuger

**Affiliations:** ^1^Department of Psychology, University of Bamberg, Bamberg, Germany; ^2^Department of Social Monitoring and Methodology, German Youth Institute, Munich, Germany; ^3^Department of Psychology, LMU Munich, Munich, Germany

**Keywords:** preschoolers, toddlers, home learning environment (HLE), digital media and learning, socio-emotional competencies, academic competencies

## Abstract

We analyzed the association between the analog and the digital home learning environment (HLE) in toddlers’ and preschoolers’ homes, and whether both aspects are associated with children’s social and academic competencies. Here, we used data of the national representative sample of Growing up in Germany II, which includes 4,914 children aged 0–5 years. The HLE was assessed via parental survey that included items on the analog HLE (e.g., playing word games, reading, and counting) and items on the digital HLE (e.g., using apps or playing with apps). Children’s socio-emotional, practical life skills, and academic competencies were assessed via standardized parental ratings. Our results indicate that there are two dimensions of the HLE, an analog and a digital, that are slightly positively associated, especially in the toddler age group. For toddlers, only analog HLE activities were associated with better socio-emotional outcomes and practical life skills. However, interaction effects indicate that toddlers with less frequent analog HLE activities showed better socio-emotional skills in households with more frequent digital activities. For preschoolers, digital HLE activities were associated with weaker socio-emotional skills but higher academic skills, although the analog HLE shows higher effect sizes for the academic outcomes. Our study points out that analog and digital HLE activities seem to be partly associated, but not interchangeable. Further, they seem to be important variables that can explain individual differences in young children’s socio-emotional, practical life, and academic competencies. However, digital media usage at home may also have negative effects on children’s social–emotional competencies. This association needs to be investigated further.

## Introduction

It is becoming increasingly evident that the nature of activities in the home learning environment (HLE) in the digital age of the 21st century is rapidly changing in terms of the resources available and the ways in which these resources are used in different contexts (Marsh et al., 2005). Digital media are commonplace nowadays in families, and European children grow up in media-rich homes (Chaudron et al., 2015). As children’s immediate caregivers usually interact with digital media daily, children consider digital devices as very important (Wirth et al., 2020b). Toddlers and preschoolers learn by observing their parents and by interacting with older siblings, and from an early age onward, they are in contact with a wide range of digital tools daily and imitate older family members’ usage (Wong, 2015).

On average, many 3- to 5-year-olds use digital technologies more than 30 min on weekdays and even longer during weekends and thus use computer-based and internet-based digital technologies at home on a regular basis (Palaiologou, 2016). Further, about a third of the children aged 0–3 years already participate in computer- and internet-based activities at home, regularly (Palaiologou, 2016). Given that children use digital media from an early age onwards, these tools can be utilized to support children’s competencies development.

The social context is crucial for learning, and this also applies to interactions with digital media (Buckingham, 2007). Given the changes that have taken place in our digital environment, concepts that have been developed to describe the early years HLE may need reconsideration. In this paper, we therefore investigate the association between the analog and the digital HLE and its associations with children’s social and academic outcomes from the first year of life onwards.

### The Concept of the “Analog” HLE and Its Effects on Child Outcomes

Children’s HLEs are typically described by the access to books, the frequency of reading to the children, and the availability of learning-oriented materials and toys. Further, parent–child interactions during a variety of learning opportunities within and out of the home, such as singing songs to the child, rhyming, and visiting cultural places (e.g., Bradley and Caldwell, 1984; Melhuish et al., 2008), and the quality of interactions during play or shared reading are considered important aspects of the HLE (e.g., Son and Morrison, 2010; Linberg, 2018; Tamis-LeMonda et al., 2019; Lehrl et al., 2020a). Although many research studies currently conceptualize the HLE domain-specifically according to the home literacy and/or numeracy model into aspects that capture formal and informal stimulation of language, literacy, and mathematics (e.g., Sénéchal and LeFevre, 2002; Manolitsis et al., 2013; Skwarchuk et al., 2014; Niklas et al., 2016; Lehrl et al., 2020a), there are also numerous studies that combine the different facets of the HLE into one indicator to capture the overall stimulation of the HLE (e.g., Melhuish et al., 2008; Son and Morrison, 2010; Niklas and Schneider, 2017; Kuger et al., 2019).

For instance, within the Effective Preschool, Primary and Secondary Education Project (EPPSE 3–16), an indicator of the early years HLE was developed that combined the frequency of seven educational activities, such as the frequency of shared book reading, visits to the library, playing games with numbers, teaching the child the alphabet, playing with letters, and teaching the child songs or nursery rhymes (Melhuish et al., 2008). This measure predicted preschooler’s literacy and numeracy outcomes, reading, and mathematics 2 years later (Melhuish et al., 2008), as well as second grader’s school grades (Bywater et al., 2015). Similarly, Son and Morrison (2010) found positive associations between the quality of the home environment as measured by a global indicator of the Home Observation for Measurement of the Environment Inventory (HOME, Bradley and Caldwell, 1984) and child outcomes including general cognitive ability and language. Furthermore, Niklas and Schneider (2017) found positive links between a kindergarten HLE-Measure comprising similar activities as in the EPPSE–HLE Index (Melhuish et al., 2008) and children’s literacy and math concurrent and grade 4 outcomes in a German sample. Consequently, when predicting various child outcomes, a broad HLE-Measure might serve as an economic, readily assessable alternative. This assumption is supported by findings of absent domain-specific effects of specific HLE measures on specific developmental domains when contrasted to each other (for an overview see, e.g., Lehrl et al., 2020a).

In addition to the well-documented positive effects of the HLE on language and academic outcomes, there is also evidence that HLE effects are not limited to these domains but are also important for children’s socio-emotional and self-regulation skills (Huntsinger et al., 2016; Rose et al., 2018; Wirth et al., 2019). In a longitudinal study, Rose et al. (2018) investigated the predictive role of the early HLE on children’s cooperative behavior, physical aggression, and emotional self-regulation at age 8, mainly mediated through early language competencies (see Wirth et al., 2019 for similar results). Further, intervention studies showed that enhancing the quality of the HLE also impacts on very young (i.e., 12 months old; O’Farrelly et al., 2018) and older (i.e., 4 years old; Bierman et al., 2015) children’s socio-emotional competencies.

To sum up, an extensive body of research has shown that the HLE is positively associated with children’s language, literacy, math, and socio-emotional skills development in early childhood (Anders et al., 2012; Niklas et al., 2018; Rose et al., 2018; Tamis-LeMonda et al., 2019; Lehrl et al., 2020b) and beyond (e.g., Niklas and Schneider, 2017; Rose et al., 2018; Lehrl et al., 2020a). However, against the background of the increasing use of digital media within the home, we are in need of research investigating how such “analog” HLEs are associated with “digital” HLEs and how both concepts relate to child outcomes in different age groups and to various developmental domains.

### The Concept of “Digital” HLE and Its Effects on Child Outcomes

Similar to the “analog” HLE, children’s “digital” HLEs may be described by the access and the frequency of usage as well as the quality of assistance/support while interacting with digital tools in the family context. This includes the access to and frequency of using, for instance, electronic toys and touchscreen devices and the parental stimulation within such contexts. In addition to e-books, digital game-based learning, which uses the entertaining power of digital games, serves an educational purpose, such as teaching math or language (All et al., 2016). An explosion in available e-books and apps has been noted over the last couple of years, especially for young children, and the majority of top-selling paid apps in 2011 were targeted for young children (Judge et al., 2015).

Although there is widespread concern that time spent with screen media replaces more traditional forms of learning (e.g., Cristia and Seidl, 2015), other researchers point out that digital technologies should be viewed as being complimentary to other resources, rather than alternatives or in competition with traditional modalities (Yelland, 2018). Through employing animated images and sound effects, digital technologies provide new and interesting experiences to the child that might motivate children more than analog tools to participate in learning opportunities (Hirsh-Pasek et al., 2015). Clearly, the HLE is an important space where digital literacy can be both employed and cultivated, and thus children’s learning and development can be supported (Meyers et al., 2013).

Actually, young children’s usage of touchscreen tablets is positively associated with emergent literacy, print awareness, print knowledge, and sound knowledge (Neumann, 2016). Further, digital media can be adapted more easily to match children’s needs and interests concerning content selection and text layout (Biancarosa and Griffiths, 2012). Consequently, in many countries, a higher number of digital devices in households coincide with better reading skills in children (Mullis and Martin, 2017). According to these findings, a digital HLE offers new possibilities to support children’s literacy development and reflects the current convergence of literacy and multimedia skills (Wirth et al., 2020a). In addition, Korat and Shneor (2019) showed that joint mother–child e-book reading compared with independent e-book reading is more effective for children’s receptive and expressive word learning. Consequently, it is preferable for young children not to use digital media alone or only passively as digital media cannot act as a substitute for human interaction (see also Hirsh-Pasek et al., 2015). However, in Germany, where this study was conducted, parents in only every second household supervise their (preschool-aged) children’s use of digital media (Marci-Boehncke et al., 2012).

Similarly, research has shown that digital learning tools can support the development of children’s numeracy competencies when used together with parents. For instance, Berkowitz et al. (2015) used an iPad app to deliver short numerical story problems to first graders and their parents. Compared with a reading control group, children’s mathematical achievement increased significantly.

As the quality of the analog HLE is associated with children’s socio-emotional competencies (e.g., O’Farrelly et al., 2018; Wirth et al., 2019), it is to be expected that the digital learning environment in families should also influence these competencies. However, research evidence regarding this point is mixed, and thus further studies and analyses are needed. In general, reviews suggest that screen time (TV, computer use, and video game) is not or negatively associated with children’s social skills. For instance, Ogelman et al. (2016) analyzed the association between 162 5- and 6-year-old children’s screen time and their social skills, which were rated by teachers. Their results revealed that children’s digital media usage duration had no effect on social skills.

Gómez et al. (2013) investigated the effect of collaborative learning on a single display computer on the social skills of 268 5- and 6-year-old children in 10 classrooms in a quasi-experimental design. The control group followed the collaborative planned activities based on the national kindergarten curriculum in an analog way. In the experimental group, children engaged in collaborative activities in a computer classroom twice each week for a period of 4 months. The activities included exchange, sort, and roleplay applications. The control and experimental groups did not differ concerning the content of activities. The experimental group showed significantly greater scores on social skills than the control group (*d* = 0.51). Some further conclusions can be drawn from Radesky et al. (2016) who examined how parents use digital media to calm difficult infants/toddlers. Toddlers rated as having social–emotional difficulties were more likely to be exposed to digital media to calm down when upset than their peers without social–emotional difficulties.

To sum up, there is some evidence that digitally supported learning with very specific high-quality digital media can support children’s learning and development (e.g., Gómez et al., 2013; Berkowitz et al., 2015; Neumann, 2016). However, there is a lack of research evidence concerning the importance of the broader defined digital HLE, i.e., access and frequency of usage of digital media, for various child outcomes, and its conjunction with the analog HLE in this context.

## The Present Study

With the widespread use of tablets and smartphones, children have increasing opportunities for interacting and learning *via* electronic devices. Unfortunately, research has not kept up with the speed of the spread of digital media-based interactions during childhood. Although research on preschool-aged children’s digital media use is increasing to some extent, studies of infant and toddlers’ digital media use are still rare (Chaudron et al., 2015). Consequently, research is needed to find out whether early shared use of digital media might harm or foster children’s development across various domains and whether digital media interactions replace traditional interactions between parents and children that have been effective in supporting children’s learning in the past century.

The present study investigates the following:

(1) The frequency of shared digital media activities, also referred to as digital HLE, in the home from an early age on, to discover the prevalence of shared digital media use in the home,
(2) How these aspects of digital HLE are associated with the analog HLE, to investigate a possible shift from analog to digital home learning environmental activities,
(3) How digital HLE and analog HLE are associated with concurrent child academic and socio-emotional outcomes, and
(4) Whether possible beneficial or harmful effects of early digital HLE are moderated by analog HLE or children’s language/practical life skills.

We assume that the analog and the digital HLE can be differentiated in two independent facets that are only slightly correlated and thus represent two distinct facets of the HLE. We furthermore assume that the frequency of digital HLE increases with age. We expect the effects of the analog and digital HLEs to be more pronounced regarding academic skills, whereas the digital HLE might have a stronger impact on socio-emotional skills, as children could be more confronted with exercises, e.g., in literacy apps.

## Materials and Methods

### Sample

We use data of the national representative sample of Growing up in Germany (AID:A II; Bien et al., 2015). The AID:A II study was carried out between 2013 and 2015 and assessed information on over 20.000 persons up to the age of 32 years (*N* = 22.424). The sample includes persons who had already taken part in the AID:A I study, a representative register-based survey, which was conducted in 2009, and a register-based refreshment sample (response rate: 34.2%). For our analyses, we used data of children aged 0 to 5 years (before school enrollment) and split them into two age-dependent samples, as we were a) interested in the differences between the very early (toddler) and the later HLE (preschool) of young children, and as b) children’s competencies and skills were assessed age-specifically. Sample 1, the “toddler sample,” includes all children at the age of 11–46 months (*n* = 2,637), and sample 2, the “preschool sample”, includes all children at the age from 47 to 71 months (*n* = 1,399).

### Measures

#### Indicators of the HLE: Analog and Digital Activities

Information on analog as well as digital media activities in the HLE was derived from an interview in which parents indicated the frequency of joint activities of the child and the parent or other persons in the household on a six-point scale, ranging from (1) never to (6) daily. The “analog HLE activities” indicator includes the mean of 11 items (e.g., reading to the child, counting, playing with alphabet toys, attending cultural activities, singing; α_*toddler*_ = 0.70, α_*preschool*_ = 0.67). “Digital HLE activities” indicator was assessed with three items by asking the parents about the frequency of joint digital media-related activities (i.e., looking at/playing with apps, going online, doing something with the computer; α_*toddler*_ = 0.67, α_*preschool*_ = 0.71). These three items capture a very general assessment of the shared use of digital media in the home environment.

#### Child Functioning

Children’s competencies and skills were assessed age-specifically for the toddler and preschool age groups.

In the toddler age group, socio-emotional as well as life skills were assessed using a selection of age-specific items derived from the monitoring of child development within the health screening of pediatricians (Petermann and Macha, 2003). Here, parents were asked to report whether the described behavior is true for the child (0 = no,1 = yes). These values were classified into categories, which were generated age-specifically within a 2-month interval: (2) maximum to smaller than one standard deviation from the mean, (1) one to under two standard deviation from the mean, and (0) two or more standard deviations from the mean. An index of practical life skills was summed up across eight items, and another set of eight items make up an index for socio-emotional development. *Practical life skills* for children at the age of 10 months included items such as pointing to an object in order to get the parent’s attention or removing barriers in order to reach an object or speaking double-sounds (such as baba, dada). *Socio-emotional development* at the age of 24 months included aspects such as displaying signs of joy when another child appears, if the child responds to a calmly spoken “no,” and if the child can be quickly calmed in everyday irritations.

In the group of preschool children, socio-emotional as well as domain-specific skills were also assessed *via* parent report. Socio-emotional skills were captured using the Strength and Difficulties Questionnaire (SDQ; Klasen et al., 2003).

Parents reported on five items about the children’s *prosocial behavior*, e.g., if the child is considerate of other people’s feelings or shares readily with other children (treats, toys, pencils, etc.), ranging from (0) not true to (2) certainly true. All five items were summed up (α_*preschool*_ = 0.62). Additionally, the four subscales on *total difficulties*, including 20 items^1^ covering emotional problems, conduct problems, hyperactivity/inattention, and peer problems, were summed up to build another index. Items referred to whether the child often seems to be worried; often has tantrums or hot tempers; is restless, overactive, and fidgety; is rather solitary; or tends to play alone were captured with the same procedure (α_*preschool*_ = 0.74).

Domain-specific skills in the age group of the 3–5-year-olds were assessed using items of the TIMMS/IGLU 2011 study (Wendt et al., 2016), which capture language as well as math skills. Parents were asked to indicate their child’s level of ability on a four-point scale, ranging from (1) not at all to (4) very good. Language skills comprise six items, such as recognizing letters, reading some words, reading sentences, writing letters, and writing some words (α_*preschool*_ = 0.78), whereas math skills capture abilities, such as counting, recognizing numbers from 0 to 10, simple summation, and simple subtraction within five items (α_*preschool*_ = 0.80).

#### Indicators of Child and Family Background

As indicators for socio-economic background of the family, we included the number of siblings living in the household, the weighted household income (OECD, 2013), the highest socio-economic status, measured *via* the International Socio-Economic Index of Occupational Status (HISEI; Ganzeboom et al., 1992), as well as the highest educational level in the household. Here, we used the CASMIN-classification (Comparative Analysis of Social Mobility in Industrial Nations; Müller et al., 1989), which contains information on school and vocational training certificates, ranging from 1 to 8 with (1) indicating general elementary education, (4) secondary school leaving certificate with vocational training, and (8) higher tertiary education (university degree). As child background information, children’s age (in months) and their gender (0 = girl, 1 = boy) were considered. Additionally, we controlled for “positive parenting” as an omnibus indicator for the socio-emotional support within the family. Parents answered four questions, such as if they praise their child or comfort their child if he/she is sad. For parents of children aged 24 months, two additional items were presented, such as “I talk to my child about what he/she has experienced” (α = 0.58).

#### Tendency of Agreement

Acquiescence has long been known to influence survey data. A particular thread to the data’s validity is differential effects depending on the respondent’s education, age, or gender (O’Muircheartaigh et al., 1999). All data used for this study were collected in a phone-based survey (CATI) with parents. In order to prevent our analyses to be corroborated by such a response bias, all multivariate analyses include a correction factor “tendency to agree.” For this, we built a ratio “tendency to agree” by dividing the number of agreeing responses across all six-point rating scales throughout the full questionnaire by the number of all valid responses. Descriptive statistics for all variables included are provided in Tables 1, 2.

**TABLE 1:** Descriptive statistics for the toddler age group.

	**Mean**	***SD***	**Min**	**Max**
Analog HLE	3.82	0.78	1	5.82
Digital HLE	1.67	0.96	1	6
Practical life skills	5.69	1.64	0	8
Socio-emotional development	6.64	1.14	1	8
Positive parenting	3.72	0.30	1.50	4
Income	1,875.6	1,675.3	3.33	47,617.1
HISEI	63.8	18.6	14.2	89.0
Education (CASMIN)	6.47	1.82	0	8
Siblings	1.93	0.90	1	13
Age in months	27.4	10.6	11	46
Gender (0 = girl, 1 = boy)	0.52	0.50	0	1
Tendency of agreement	0.51	0.075	0.28	0.96

**TABLE 2:** Descriptive statistics for the preschool age group.

	**Mean**	***SD***	**Min**	**Max**
Analog HLE	4.20	0.60	2.18	5.73
Digital HLE	2.16	1.07	1	6
SDQ: prosocial behavior	8.18	1.61	1	10
SDQ: total difficulties	7.27	4.40	0	26
Language skills	2.93	0.64	1	4
Math skills	2.08	0.68	1	4
Positive parenting	3.64	0.31	2	4
Income	1,968.5	1,683.6	115.4	33,333.3
HISEI	63.0	18.7	14.2	89.0
Education (CASMIN)	6.44	1.77	0	8
Siblings	2.16	0.91	0	12
Age in months	58.3	6.15	48	71
Gender (0 = girl, 1 = boy)	0.52	0.50	0	1
Tendency of agreement	0.50	0.08	0.21	0.79

### Analytic Strategy

First, to examine whether the analog and the digital HLE can be differentiated into two separate facets of the HLE, we conducted confirmatory factor analyses and used chi-square difference testing to decide on model selection. Second, we explored (a) the frequency of shared digital media use by investigating the proportion of families sharing digital media at least seldom with their child, as an indicator of having overall shared contact with digital devices at several ages (the age groups were split to get a better impression of the increase across ages within the toddler and preschool age groups) and (b) the relation between analog and digital HLEs using bivariate correlations. Third, the associations between digital and analog HLEs with child outcomes were analyzed using multiple regression models for both age groups. Here, we included the tendency of agreement to capture the variance between HLE and child outcomes that can be attributed to a general tendency of agreement to different items. Note that we did not control for child age in the toddler age group years as the used instrument (Petermann and Macha, 2003) comprises age-specific items and thus age-specific practical life skills as well as socio-emotional development (2-month intervals) were used in the analyses.

Fourth, to analyze whether the analog HLE moderates the effects of the digital HLE, we included interaction terms for all regression models. Furthermore, for the models with the socio-emotional skills as outcomes, we analyzed whether practical life skills (toddlers) or language skills (preschoolers) moderate the effects of the digital HLE by including interaction terms into the regression models. To visualize the interaction effects, we plotted predictive margins in which we only visualized results for low (mean −1 SD), medium (mean), and high (mean +1 SD) value of the respective scale, for visual clarity. Interaction effects were plotted when *p* was smaller than 0.10. All models were run using Stata 15.

## Results

### Digital and Analog HLE: Frequencies and Internal Associations

We first explored whether digital and analog HLEs can be differentiated into two separate dimensions. We ran confirmatory factor analyses to decide whether a one-factor solution or the proposed two factor solution would fit the data better. Results of chi^2^ difference testing were in favor of the two-factor model (Δχ^2^ = 2,380.734, *df* = 1, *p* < 0.001). In addition, the correlational analyses (Tables 3, 4) showed only small correlations between the two dimensions.

**TABLE 3:** Bivariate correlations of the study variables in the toddler age group.

	**1**	**2**	**3**	**4**	**5**	**6**	**7**	**8**	**9**	**10**
Analog HLE	1.00									
Digital HLE	0.19***	1.00								
Practical life skills	0.21***	0.07***	1.00							
Socio-emotional development	0.19***	0.01	0.42***	1.00						
Positive parenting	0.03^+^	−0.09***	0.10***	0.07***	1.00					
Income	−0.03^+^	0.01	0.03	0.06**	0.04*	1.00				
HISEI	0.02	0.02	0.04*	0.05**	0.01	0.26***	1.00			
Education (CASMIN)	0.03	0.07**	0.03	0.07***	0.01	0.24***	0.74***	1.00		
Siblings	–0.01	−0.09***	0.01	0.03^+^	−0.13***	−0.06**	–0.02	−0.04*	1.00	
Age in months	0.35***	0.28***	0.18***	0.16***	−0.25***	0.00	0.05**	0.05*	0.13***	1.00
Gender (0 = girl, 1 = boy)	−0.06**	–0.01	−0.07***	−0.10***	–0.01	0.01	0.02	0.01	–0.01	–0.01

n = 2,637; ^+^p < 0.10, *p < 0.05, **p < 0.01, ***p < 0.001; controlled for tendency of agreement.

**TABLE 4:** Bivariate correlations of the study variables in the preschooler age group.

	**1**	**2**	**3**	**4**	**5**	**6**	**7**	**8**	**9**	**10**	**11**	**12**
Analog HLE	1.00											
Digital HLE	0.05^+^	1.00										
SDQ: prosocial behavior	0.08**	−0.07**	1.00									
SDQ: total difficulties	−0.07**	0.08**	−0.31***	1.00								
Language skills	0.19***	0.16***	0.12***	−0.16***	1.00							
Math skills	0.20***	0.14***	0.16***	−0.15***	0.65***	1.00						
Positive parenting	0.18***	–0.03	0.16***	−0.18***	0.06*	0.01	1.00					
Income	0.04^+^	0.04	−0.05*	−0.09**	0.10***	0.09**	0.04	1.00				
HISEI	0.09***	0.02	–0.02	−0.19***	0.17***	0.11***	0.01	0.28***	1.00			
Education (CASMIN)	0.11***	0.05^+^	–0.01	−0.20***	0.17***	0.12***	0.01	0.22***	0.68***	1.00		
Siblings	−0.09***	−0.10***	0.00	−0.05^+^	−0.07**	−0.05*	−0.11***	−0.06*	0.06*	0.02	1.00	
Age in months	–0.02	0.04	0.08**	−0.08**	0.50***	0.39***	–0.02	0.03	0.11***	0.06**	0.01	1.00
Gender (0 = girl, 1 = boy)	−0.07**	0.03	−0.15***	0.14***	–0.03	−0.23***	–0.01	0.00	0.01	0.01	–0.00	–0.00

n = 1,399; ^+^p < 0.10, *p < 0.05, **p < 0.01, ***p < 0.001; controlled for tendency of agreement.

As depicted in Table 5, shared digital media activities increased across age, in particular concerning the shared use of a computer. However, about 15% of children in the sample had experiences with sharing digital media already in their toddler years.

**TABLE 5:** Proportion of shared digital media activities in the home (at least seldom).

	**Age of child in years**						
	**<1**	**1**	**2**	**3**	**4**	**5**	**6**
Overall digital HLE	14.7	30.3	57.4	67.8	72.6	83.0	85.2
Sharing apps	5.4	18.1	34.3	39.5	45.9	49.5	45.0
Using the internet	5.4	10.1	25.8	39.4	48.3	56.4	64.9
Doing something with the computer	9.0	20.0	40.7	49.5	58.4	69.4	75.1

**Digital HLE, percentage of children who are at least seldom involved in either sharing apps, using the internet, or doing something with the computer*.*

### Relations Between HLE and Child Outcomes and Potential Moderators

**FIGURE 1:**
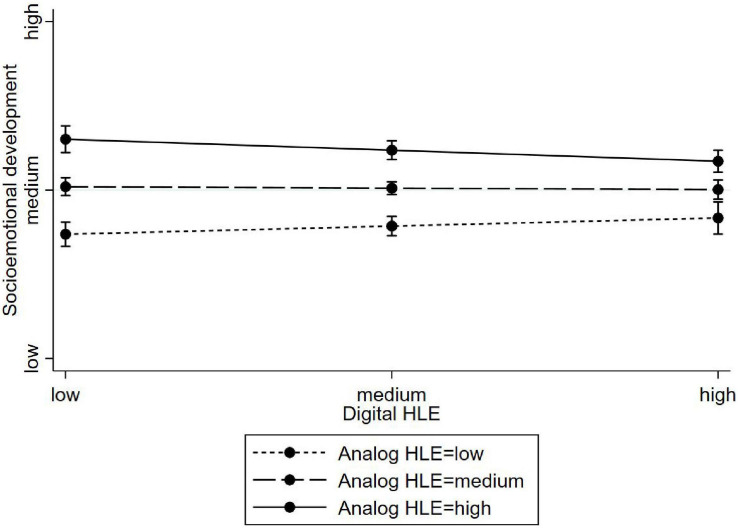
Moderating effects of analog HLE on the association between digital HLE and socioemotional development (toddler age group).

As can be seen in Table 6, toddler’s socio-emotional skills were associated with analog HLE (β = 0.23, *p* < 0.001), but not with digital HLE (β = −0.03, ns) (Model 1). A similar pattern is visible for the association of practical life skills (Model 4, analog HLE: β = 0.24, *p* < 0.001; digital HLE: β = 0.03, *p* < 0.10). In addition, the interaction between digital and analog HLEs was significant for socio-emotional skills (Model 2, β = −0.34, *p* < 0.01). As depicted in Figure 1, digital HLE moderates the effect of analog HLE in that way, that socio-emotional skills increase for children with low analog HLE when being involved in more digital HLE. No other interaction terms were significant (Models 3 and 5).

**TABLE 6:** Multivariate regressions: associations between toddler’s socio-emotional outcomes, practical life skills, and digital and analog HLEs.

	**Model 1**	**Model 2**	**Model 3**	**Model 4**	**Model 5**
	**Socio-emotional**	**Socio-emotional**	**Socio-emotional**	**Practical life**	**Practical life**
	**development**	**development**	**development**	**skills**	**skills**

Analog HLE	0.23***	0.33***	0.14***	0.24***	0.28***
Digital HLE	–0.03	0.27**	−0.14*	0.03^+^	0.18^+^
Positive parenting	0.07***	0.07***	0.03	0.10***	0.10***
Income	0.05**	0.05**	0.04*	0.03	0.03
HISEI	–0.02	–0.02	−0.04^+^	0.05	0.05
Education (CASMIN)	0.08**	0.08**	0.09***	–0.02	–0.02
Siblings	0.04*	0.04*	0.03^+^	0.03	0.03
Gender (0 = girl, 1 = boy)	−0.09***	−0.09***	−0.07***	−0.06**	−0.06**
Tendency of agreement	0.03	0.03	0.02	0.02	0.02
HLE media activities × HLE educational activities		−0.34**			–0.17
Practical life skills			0.34***		
HLE media activities × practical life skills			0.11		
Observations	2,637	2,637	2,637	2,637	2,637
*R*^2^	0.08	0.08	0.22	0.08	0.08
Adjusted *R*^2^	0.08	0.08	0.21	0.08	0.08

Standardized beta coefficients, ^+^p < 0.10, *p < 0.05, **p < 0.01, ***p < 0.001.

**FIGURE 2:**
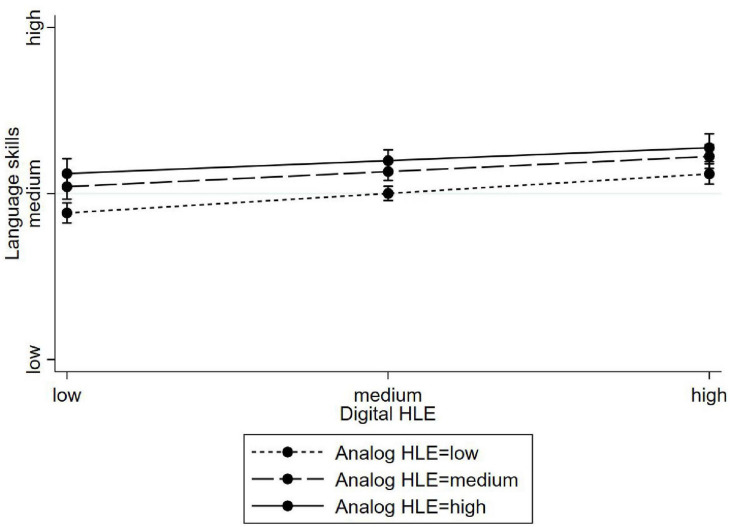
Moderating effects of analog HLE on the association between digital HLE and language skills (preschooler age group).

Table 7 shows the results for the preschoolers, that differ slightly: While the analog HLE is positively associated with prosocial behavior (Model 6, β = 0.06, *p* < 0.05) but not with total difficulties (Model 9, β = −0.02, ns), a greater experience of digital HLE goes along with less socio-emotional skills (Models 6 and 9, prosocial behavior: β = −0.06, *p* < 0.001; total difficulties: β = 0.07, *p* < 0.01). No significant interaction terms were found (Models 7, 8, 10, and 11). For parent-rated academic skills (see Table 8), we found positive associations of both, the analog and the digital HLE with language (Model 12) and math skills (Model 14). Here, the effects were more pronounced for the analog HLE. For language skills, we found a small moderator effect (Model 13). For children with high analog HLEs, the digital HLE makes no difference for their language skills. However, children with low analog HLE showed greater language skills when experiencing a higher level of digital HLE (Figure 2). No significant interaction terms were found for math skills (Model 14).

**TABLE 7:** Multivariate regressions: associations between preschooler’s socio-emotional outcomes and digital and analog HLEs.

	**Model 6**	**Model 7**	**Model 8**	**Model 9**	**Model 10**	**Model 11**
	**SDQ: prosocial**	**SDQ: prosocial**	**SDQ: prosocial**	**SDQ: total**	**SDQ: total**	**SDQ: total**
	**behavior**	**behavior**	**behavior**	**difficulties**	**difficulties**	**difficulties**

Analog HLE	0.06*	0.03	0.04	–0.02	–0.02	0.01
Digital HLE	−0.06*	–0.17	−0.20^+^	0.07**	0.08	0.26*
Positive parenting	0.15***	0.15***	0.14***	−0.18***	−0.18***	−0.17***
Income	−0.06*	−0.06*	−0.06*	–0.03	–0.03	–0.03
HISEI	–0.03	–0.03	–0.04	−0.08*	−0.08*	−0.08*
Education (CASMIN)	0.02	0.02	0.01	−0.13***	−0.13***	−0.12***
Siblings	0.01	0.01	0.02	−0.06*	−0.06*	−0.06*
Age in months	0.10***	0.10***	0.05	−0.06*	−0.06*	0.00
Gender (0 = girl, 1 = boy)	−0.14***	−0.14***	−0.14***	0.14***	0.14***	0.13***
Tendency of agreement	0.19***	0.19***	0.19***	−0.08**	−0.08**	−0.08**
Analog HLE × digital HLE		0.12			–0.01	
Language skills			0.04			–0.06
Digital HLE × language skills			0.15			–0.20
Observations	1,399	1,399	1,399	1,399	1,399	1,399
*R*^2^	0.13	0.13	0.13	0.11	0.11	0.13
Adjusted *R*^2^	0.12	0.12	0.13	0.11	0.11	0.12

Standardized beta coefficients, ^+^p < 0.10, *p < 0.05, **p < 0.01, ***p < 0.001.

**TABLE 8:** Multivariate regressions: associations between preschooler’s academic outcomes and digital and analog HLEs.

	**Model 12**	**Model 13**	**Model 14**	**Model 15**
	**Language**	**Language**	**Math**	**Math**
	**skills**	**skills**	**skills**	**skills**

Analog HLE	0.20***	0.28***	0.21***	0.26***
Digital HLE	0.12***	0.40**	0.11***	0.27^+^
Positive parenting	0.03	0.03	–0.02	–0.02
Income	0.04^+^	0.04^+^	0.05*	0.05*
HISEI	0.02	0.02	–0.01	–0.01
Education (CASMIN)	0.09**	0.09**	0.07*	0.07*
Siblings	−0.04^+^	−0.04^+^	–0.03	–0.03
Age in months	0.49***	0.49***	0.39***	0.39***
Gender (0 = girl, 1 = boy)	–0.02	–0.03	−0.22***	−0.22***
Tendency of agreement	–0.03	–0.03	0.02	0.01
Analog HLE × digital HLE		−0.30^+^		–0.17
Observations	1,399	1,399	1,399	1,399
*R*^2^	0.33	0.33	0.28	0.28
Adjusted *R*^2^	0.32	0.33	0.27	0.27

Standardized beta coefficients, ^+^p < 0.10, *p < 0.05, **p < 0.01, ***p < 0.001.

## Discussion

The aim of the present study was to investigate a potentially new dimension of the HLE, i.e., a digital HLE, that includes the frequency of shared digital media activities from an early age on, its association with analog HLE, and how these two dimensions of the HLE are related to children’s socio-emotional, practical life, and academic outcomes at toddler and preschool age. Furthermore, we explored whether potentially beneficial or harmful effects of digital HLE are enhanced or compensated by an analog HLE or by children’s language/practical life skills. Our results indicate that digital and analog HLEs can be seen as separate dimensions that are only marginally related to each other and differentially related to children’s outcomes.

The positive correlations between digital and analog HLEs in the toddler age group show that parents who actively involve their children in educational “analog” activities in this early phase of development do so with digital media, too. This finding aligns with previous research that showed that parents of children below the age of 3 years who read more often to their children also tended to show a greater frequency in various other activities, such as singing, playing with dolls, and doing crafts (Wirth et al., 2020b). We found that children’s digital HLE activities rapidly increase with age. However, in the older age group, digital and analog HLEs were no longer associated. Consequently, there seems to be neither a shift from an analog to a digital HLE, which would have been indicated by a negative correlation, nor a hint to a general indicator of the HLE comprising analog and digital aspects, which would have been indicated by a high, positive correlation. Rather, families’ HLE seems to follow differential developmental patterns, as their children grow older. Therefore, as shown in many studies before, the HLE is no unitary construct and needs to be differentiated (e.g., Skwarchuk et al., 2014; Wirth et al., 2019; Lehrl et al., 2020a). The present study gives further evidence that the medium through which stimulation in the home takes place is another variable that distinguishes dimensions of the HLE.

This differentiation gains further importance when inspecting the differential association between the digital and analog HLEs and child outcomes in the two age groups. In the younger age group, analog HLE activities were important for explaining variance in self-reported socio-emotional and practical life skills, whereas digital HLE activities were not. However, moderation analyses revealed that children with less frequent analog HLE activities showed greater socio-emotional skills when experiencing more frequent digital activities. Research has shown that low stimulating (analog) HLEs might be a risk factor for children’s developing academic and social skills (e.g., Mistry et al., 2008, 2010). Consequently, for children being at risk in terms of their low stimulating analog HLEs, sharing digital media may bring parents and children together in meaningful interactions that in turn may protect children from unfavorable developmental trajectories. There are hints that low socioeconomic status (SES) families are more involved in the education of their children when using digital tools, such as electronic books or apps (e.g., Erdogan et al., 2019; Wirth et al., 2020a). However, one has to bear in mind that we measured the digital HLE as the frequency of parent–child interaction when sharing digital devices, and not, for instance, as the frequency of the child passively watching TV alone.

In the preschooler’s age group, however, digital HLE activities were associated with weaker self-reported socio-emotional skills. Although the effect sizes are small, these results are alarming, especially when considering the non-significant moderator effects, which indicate that the negative digital HLE effect cannot be compensated by a high-quality analog HLE. As children grow older, interactions with digital media might be less communicative or guided by parents than the same interactions conducted with toddlers. For instance, a Korean study with 5-year-old children showed that an earlier onset of computer usage was associated with longer computer usage later. In addition, children with an early onset were more likely to play computer games and were less likely to be supervised while using the computer, which, in turn, was associated with higher scores on internet addiction and lower scores in socio-emotional competencies (Seo et al., 2011). Contrary to these findings, Gómez et al. (2013) reported positive effects of collaborative activities around computers on children’s social development skills. Obviously, the social and emotional effects of shared or non-shared digital media use on young children are underexplored, and more research is needed, especially in light of the often-proclaimed negative effects (e.g., Cordes and Miller, 2000; Sigman, 2012; Fröhlich-Gildhoff and Fröhlich-Gildhoff, 2017) and the resulting possible lost resources that may instead foster academic skills (Plowman and McPake, 2013).

Although only used as a control variable, our analyses show that the effects of overall warm and responsive interactions as indicated through the variable “positive parenting” compared with the digital HLE were twice as large (Tables 6, 7) and seemed to be more meaningful in the development of socio-emotional skills of children (see also Thomas and Zimmer-Gembeck, 2007 for an overview on the effects of positive-parenting interventions). The digital HLE was significantly associated with academic skills, although the analog HLE showed higher effect sizes. Concerning vocabulary acquisition, some studies also reported positive effects of digital media use, yet the greatest effects were observed when such use was guided by adults (Teepe et al., 2016; Walter-Laager et al., 2016). In addition, a meta-analysis showed that animated pictures as additions to stories can boost vocabulary development when they are congruent to the story (Takacs et al., 2015). Similar improvements have been shown in early number skills after interacting with math apps (Mattoon et al., 2015; Schacter and Jo, 2016; Watts et al., 2016). Consequently, our research is in line with the huge body of results that emphasizes the role of the analog HLE in shaping children’s language and mathematical skills (e.g., Hindman and Morrison, 2012; Niklas and Schneider, 2017; Tamis-LeMonda et al., 2019; Lehrl et al., 2020a,b). In addition, the present study adds to our knowledge by showing that the digital HLE adds to the effects of analog HLE activities.

However, the following limitations mark this study: HLE activities as well as child’s competencies were reported by parents, and although they are based on established measures often used in other studies (such as the SDQ), they might be biased. Although we tried to diminish this effect by controlling for the general tendency of agreement, we cannot completely rule this possibility out.

Furthermore, our analyses are only cross-sectional; thus, we do not know the direction of effects and no causal claims can be made. Potentially, child’s competence level may influence the frequency of digital HLE activities and not the other way around (Hygen et al., 2019). Indications of such associations are mainly found in the area of socio-emotional competences. For example, parents might react to social–emotional difficulties by trying to calm difficult infants/toddlers with digital media activities (Radesky et al., 2016). To disentangle such complex interrelations and the impact of both digital and analog HLEs on the development of socio-emotional and academic skills is certainly an avenue for future research. Furthermore, the data of our study are representative for families in 2013. The rapid growth in the digital sector in the last years underlines the need for further research in this area to investigate possible drifts and changes regarding the analog and digital HLEs.

Additionally, it must be considered that our digital HLE measure was a very general assessment of the shared use of digital media in the home environment that consists of only three items, and that we have no information on the content of the digital media activities and the quality of the interaction between parent and child during shared digital media use. Some study results point out that parent–child interaction and parents’ talk may be impoverished when they use digitally enhanced books (Zosh et al., 2015). However, as already mentioned, digital media might also foster children’s learning (e.g., Takacs et al., 2015), but only if it is designed appropriately (Hirsh-Pasek et al., 2015). For example, Parish-Morris et al. (2013) examined and compared interactions of parents with their 3-year-old children with digital books, containing different enhanced features (e.g., pre-recorded sounds), and found more enhanced features to be less beneficial for the parent’s use of high-quality language (dialogic reading strategies) and the child’s learning (story comprehension). This finding might even be true for younger children. Sosa (2016) demonstrated in a controlled experiment that in the condition with electronic toys, both adults and children produced fewer words, and conversation turns occurred less often than parent–child play with traditional toys and books.

Therefore, more research is needed to understand under which circumstances digital devices and apps may benefit children’s development. Here, policy for children’s media as well as research should also move away from focus on “screen time” and provide parents with specific guidelines to select quality content to optimize media experiences for young children (Huber et al., 2018). Detailing children’s screen media experience in a digital HLE will provide a better understanding of whether digital media can be used to promote learning in young children.

## Conclusion

Children’s experiences with technology and interactive digital media are increasingly a part of their daily lives. With the present study, we have shown that sharing digital media at home may be seen as the digital facet of the HLE, which can be differentiated from the analog HLE. Our findings show once more the important role of the analog HLE for children’s competencies, but extend research by showing that the digital HLE also affects aspects of children’s development. Digital HLE means that children and parents share digital devices, and that parents are actively involved. Thus, here, parents are clearly the most important partners for young children’s interaction with digital technologies, and it is to be expected that the effect of digital media will depend on parents’ choice of suitable media and the support of their children. Useful support for parents in deciding how children can best benefit from digital technologies might be needed.

## Data Availability Statement

Publicly available datasets were analyzed in this study. Data can be retrieved under this URL: https://surveys.dji.de.

## Ethics Statement

Ethical review and approval was not required for the study on human participants in accordance with the local legislation and institutional requirements. Written informed consent to participate in this study was provided by the participants’ legal guardian/next of kin.

## Author Contributions

SL and AL developed the research ideas, the constructs, and were responsible for the analyses. SL wrote the first draft of the manuscript. FN and SK contributed to the theoretical and discussion section and revised the manuscript. All authors contributed to the article and approved the submitted version.

## Conflict of Interest

The authors declare that the research was conducted in the absence of any commercial or financial relationships that could be construed as a potential conflict of interest.

## Footnotes

1 Please note that as the SDQ version for children from the age of 4–17 years was used, two items (“lies” and “steals”) were only answered if children were 48 months or older.
